# Retinal Arteriolar Wall Remodeling in Diabetes Captured With AOSLO

**DOI:** 10.1167/tvst.12.11.16

**Published:** 2023-11-14

**Authors:** Kaitlyn A. Sapoznik, Thomas J. Gast, Alessandra Carmichael-Martins, Brittany R. Walker, Raymond L. Warner, Stephen A. Burns

**Affiliations:** 1 School of Optometry, Indiana University, Bloomington, IN, USA; 2 College of Optometry, University of Houston, Houston, TX, USA; 3 Scheie Eye Institute, Department of Ophthalmology, University of Pennsylvania, Philadelphia, PA, USA

**Keywords:** adaptive optics, vascular remodeling, diabetes

## Abstract

**Purpose:**

Adaptive optics scanning laser ophthalmoscopy (AOSLO) enables the visualization and measurement of the retinal microvasculature structure in humans. We investigated the hypothesis that diabetes mellitus (DM) induces remodeling to the wall structure in small retinal arterioles. These alterations may allow better understanding of vascular remodeling in DM.

**Methods:**

We imaged retinal arterioles in one eye of 48 participants (26 with DM and 22 healthy controls) with an AOSLO. Structural metrics of 274 arteriole segments (203 with DM and 71 healthy controls) ≤ 50 µm in outer diameter (OD) were quantified and we compared differences in wall thickness (WT), wall-to-lumen ratio (WLR), inner diameter (ID), OD, and arteriolar index ratio (AIR) between controls and participants with DM. We also compared the individual AIR (iAIR) in groups of individuals.

**Results:**

The WLR, WT, and AIRs were significantly different in the arteriole segments of DM participants (*P* < 0.001). The iAIR was significantly deviated in the DM group (*P* < 0.001) and further division of the participants with DM into groups revealed that there was an effect of the presence of diabetic retinopathy (DR) on the iAIR (*P* < 0.001).

**Conclusions:**

DM induces remodeling of wall structure in small retinal arterioles and in groups of individuals. The use of AIR allows us to assess remodeling independently of vessel size in the retina and to compute an index for each individual subject.

**Translational Relevance:**

High-resolution retinal imaging allows noninvasive assessment of small retinal vessel remodeling in DM that can improve our understanding of DM and DR in living humans.

## Introduction

In diabetes mellitus (DM), hyperglycemia occurs and induces changes to the systemic macro and microvasculature.^1^ Diabetic retinopathy (DR) is one of the most common microvascular complications of DM^2^ and is a leading cause of blindness in working age adults in the United States and globally.^3^ DR is not diagnosed clinically until retinal lesions are observed with clinical ophthalmoscopy and/or retinal imaging. However, subclinical microvascular changes, like pericyte loss and wall thickening, may precede clinically detectable DR. Continued improvement in the resolution of in vivo retinal imaging allows for many of these changes to be investigated as potential biomarkers for both retinal and systemic diseases.^4^^,^^5^ Quantification of the wall and lumen structure of the retinal arterioles in living humans is an important candidate biomarker that warrants further attention^5^ especially in the setting of DM and DR.

Retinal and systemic small vessels, often referred to as resistance vessels, are the sites of blood flow autoregulation.^6^^,^^7^ These vessels are susceptible to structural remodeling in vascular diseases like DM.^6^^–^^8^ Such remodeling includes changes to the smooth muscle cells of the vascular walls that may be accompanied by constriction or dilation of the lumen. Other changes like vessel tortuosity may also be observed.^9^ Currently, two chief methods can be used to quantify vascular wall remodeling in these small vessels in living humans: (1) assessment of the anatomically measured media-to-lumen ratios (MLRs) from subcutaneous biopsies using wire micromyography, and (2) quantifying the wall and lumen structures to calculate wall-to-lumen ratios (WLRs) in retinal arterioles using retinal imaging techniques. Although these two measurements are similar, wire micromyography enables one to isolate measurements of the media of the vessel (i.e. the middle layer primarily composed of smooth muscle cells) whereas retinal imaging does not. In type 2 DM, a volumetric increase in vascular smooth muscle cells (hypertrophic remodeling) is present in subcutaneous gluteal and abdominal tissue^8^^,^^10^^–^^13^ and is amplified with concomitant hypertension (HTN).^8^ Although micromyography is precise, the procedures needed to measure vessel parameters are invasive and a biopsy is required. Despite the vessels being of larger caliber (100-350 µm) than most retinal arterioles, the WLRs of retinal arterioles, when measured with scanning laser Doppler flowmetry and Adaptive optics (AO) retinal imaging, strongly correlates with the MLRs of the subcutaneous arterioles.^14^^,^^15^ Therefore, retinal imaging may allow for the quantification of small vessel remodeling noninvasively and, furthermore, allows measurement of the vessels in vivo with active blood flow*.* AO retinal imaging provides higher resolution of the vascular structural changes by correcting for ocular aberrations and allows for the use of multiply scattered light imaging to visualize the wall and lumen of the retinal vessels even in small retinal arterioles.^16^^–^^20^ This has led to a growing literature quantifying the retinal arteriolar wall remodeling measured with AO imaging as a potential biomarker for ocular and systemic disease.^5^ In diabetes, increases in WLRs and wall thickness, also referred to as parietal thickness, are observed^21^^–^^26^ as well as alterations in arteriole branching.^27^

In this study, we used an adaptive optics scanning laser ophthalmoscope (AOSLO) to investigate the arteriolar remodeling that occurs in small retinal arterioles (less than or equal to 50 µm in outer diameter). The lateral resolution of our AOSLO system (approximately 2 µm) allows for visualization and quantification of the vascular wall.^16^ In DM and DR, capillary pericytes are lost impairing blood flow autoregulation and loss of myogenic control.^26^^,^^28^^–^^30^ Therefore, we hypothesize that smaller arterioles – located closer to the capillary bed than larger arterioles – may have structural remodeling that occurs early in disease and would be significantly remodeled in diabetes. To assess this, we quantified the structural properties of the arterioles including wall thickness, inner diameter (ID), outer diameter (OD), and WLR. Due to the covariance of WLR and vessel size,^18^ we used a novel metric, the arteriole index ratio (AIR), to determine the amount of remodeling for a given vessel. The assignment of an index to each vessel allowed us to average the AIR across vessel segments in each individual, giving an index for each participant (individual AIR [iAIR]). That is, the AIR allows estimating the extent to which structural parameters of the vessel (AIR) or vessels of an individual (iAIR) deviate from a young, control population measured in a previous study.^18^

## Methods

### Participants

All study protocols were approved by the Indiana University Institutional Review Board (IRB) and adhered to the tenets of the Declaration of Helsinki. All participants received a full explanation of the procedures and consequences of this study and signed a consent form approved by the Indiana IRB.

Adults 18 years and older were recruited for this study. Participants were included in the group with diabetes if they had a diagnosis of type 1 or 2 DM from a medical provider. Thirty-six participants with DM participated in this study. A careful medical history and clinical examination were performed and participants in the DM group were excluded if they had glaucoma, poor quality AOSLO imaging, or a history of systemic disease (other than controlled hypertension) known to cause retinopathy, or detection of retinal pathology on clinical examination other than DR. Participants included in the control group had no known ocular or systemic pathology. The exclusion criteria for control participants were the same as the participants with DM, except those with HTN and cardiovascular disease were also excluded. If imaging was performed on both eyes of a participant, only one eye was analyzed.

### Clinical Examination

A clinical examination was performed on each participant. Examinations included at a minimum: (1) visual acuity; (2) blood pressure; (3) slit lamp evaluation; and (4) measurement of intraocular pressure (iCare TA01i; iCare Finland Oy, Helsinki, Finland). Pupils were dilated with one drop each of 1% tropicamide and 2.5% phenylephrine in the study eye and a fundus examination was performed if it had been more than 12 months since the patient's last comprehensive eye examination. Axial length was measured with the IOLMaster (Carl Zeiss Meditec, Carlsbad, CA, USA). The control participants were recruited from the Indiana University School of Optometry clinic and from a local medical clinic. Experimental imaging measurements are described below.

### OCT Imaging

A 20 × 20 degree volumetric SD-OCT scan, centered on the fovea (193 b-scans, 30 µm spacing on the high speed [HS] setting) was collected at each visit. Additional volumetric scans were also obtained in some participants with DM in regions of interest (ROI) identified by previous medical records from recent examinations and/or fundus examination. In all DM participants, a 20 × 10 degree optical coherence tomography angiography (OCTA) scan (256 b-scans, 11 µm spacing, high resolution [HR] setting) that includes the fovea, parafoveal vasculature, and vasculature temporal to the fovea corresponding to the location of our AOSLO vascular imaging was obtained in participants with diabetes. All participants had a 30-degree scanning laser ophthalmoscopy (SLO) fundus image (Spectralis spectral domain-optical coherence tomography [SD-OCT]; Spectralis, Heidelberg, Germany) taken centered on the fovea. For the DM participants, nine 30-degree infrared SLO (IR SLO) images (Spectralis SD-OCT, Heidelberg, Germany) were obtained for each DM participant to generate a 90 × 60 degree montage to evaluate the mid-peripheral retina for DR lesions like neovascularization.

#### Grading of Diabetic Retinopathy

We divided our participants into broad groups of DR: (1) DM without clinically visible DR, (2) DM with nonproliferative diabetic retinopathy (NPDR), and (3) DM with proliferative diabetic retinopathy (PDR). All examination records from our research studies as well as other available clinical data were reviewed to determine the level of DR in each participant. Grading of DR on fundus examinations for participants without a clinical grade were performed by two of the authors (K.A.S., an optometrist and T.J.G., an ophthalmologist). Because the fundus examination generally preceded the AOSLO imaging used in this analysis, the IR SLO images and OCT b-scans were also used to confirm the previously graded level of retinopathy as well as to monitor changes. The IR SLO images were assessed for the presence of microaneurysms, retinal hemorrhages, cotton wool spots, exudates, intraretinal microvascular abnormalities (IRMA), venous beading, retinal neovascularization, neovascularization of the optic nerve head, and vitreous hemorrhage. OCT and OCTA images were used to confirm their presence or absence of subtle vascular lesions, such as microaneurysms in the IR SLO images.

Additionally, all b-scans collected for each participant from the standard OCT imaging session were reviewed to detect the presence of macular edema. The central subfield thickness for each participant was also collected for analysis.

Evaluation of all IR SLO images, OCT, and OCT-A scans were performed by author K.A.S. and confirmed by author T.J.G. when needed.

### AOSLO Imaging

#### Image Acquisition

AOSLO imaging was performed with the Indiana AOSLO,^19^ which generates four simultaneous images from four detection channels; one confocal and three multiply scattered light channels. The multiply scattered light images were generated from (1) a digital micromirror device (DMD) serving as a configurable aperture or (2) a 10 airy disk diameter (ADD) pinhole aperture displaced 6 ADD,^16^^,^^17^ or (3) a multimode fiber 10 to 12 ADD in diameter acting as a displaced aperture displaced approximately 6 ADD.^31^^,^^32^ For the DMD measurements, a split annulus aperture with an inner diameter at 8.3 ADD and outer diameter at 22.7 ADD^19^ was used.

To form an image, the retina was sampled with either a 1.2 degrees × 1.3 degrees or 2 degrees × 1.7 degrees field (0.67 or 1.0 µm per pixel for a standard-length eye). One hundred frames of video were collected at each location. Once collected, the imaging field was moved roughly one half the width of the imaging field size in either the horizontal or vertical direction, and image acquisition repeated by moving the imaging field. This allowed data, after processing, to be montaged. Imaging sessions for participants with DM included a protocol with both fixed ROIs (across all subjects) and a variable segment (for targeting other areas of interest). The fixed ROIs included either a 6 × 6-degree area centered over the fovea and a 4 × 6-degree ROI temporal to and contiguous with the foveal centered ROI, or a 12 × 7-degree ROI. For the fixed ROIs, our AOSLO system was focused on the superficial vascular plexus. For the targeted segment, we uploaded an IR SLO image from our OCT system to the AOSLO system and use our steerable AOSLO^33^ to evaluate areas of interest. Data for control participants were collected with various protocols used in the laboratory for vascular imaging and all images were acquired within the posterior pole.

#### Image Processing

Following each imaging session, custom MATLAB (Mathworks, Inc., Natick, MA, USA) software automatically determined a video frame with minimal eye movement distortion and then used strip alignment to align other frames to the chosen frame, removing the impact of small eye movements and excluding frames with large eye movements or blinks. The program then computed average images,^34^ aligned videos, and generated vascular perfusion maps based on the movement of blood cells.^17^

### Vessel Segment Measurements

**Figure 1.**
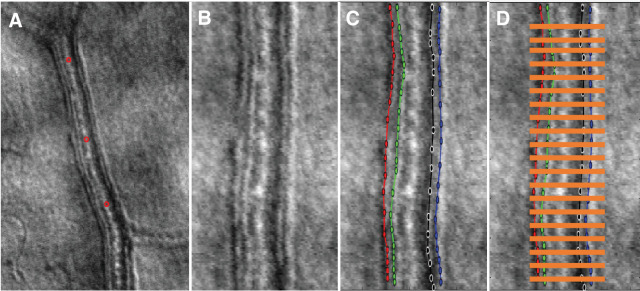
Example of vessel segment (outer diameter = 27 µm) measurement using semi-automated custom MATLAB software. Panel (**A**) Each vessel segment is selected along the center of the lumen. Panel (**B**) The image intensity along lines perpendicular to the blood vessel are calculated for each pixel along the blood vessel yielding a verticalized image. Panel (**C**) Next, the vessel inner and outer diameter edges were then traced with manual mouse clicks. Panel (**D**) Each structural parameter was computed at every five pixels along the arteriole segment length (not necessarily shown to scale). The average of these was then used in the final analysis.

All images acquired in the imaging session of the study eye were reviewed. In each participant, arteriole segments were selected for measurement when the image quality and aperture orientation allowed for clear visualization of the delineation of wall edges (border of wall to lumen and border of wall to surrounding retina) on both sides of the arteriole segment. The multiply scattered light image with the best contrast of the arteriole structure was selected for quantification.^19^ To measure the vessel structure, custom MATLAB software was developed for semi-automated grading as demonstrated in Figure 1. A segment of the arteriole that did not contain major branches was identified and the center of the vessel lumen marked (see Fig. 1A). Because vessel visualization is highly dependent on aperture orientation^16^^,^^19^^,^^35^ we reviewed each multiply scattered light image to confirm the lack of vessel branches. The image intensity along lines perpendicular to the blood vessel was calculated for each pixel along the blood vessel (see Fig. 1B). The vessel wall edges were then traced manually (see Fig. 1C) and the distances between the wall traces were automatically calculated at 5-pixel intervals (see Fig. 1D). The average wall thickness, ID, OD, and WLR were automatically computed in MATLAB.

**Figure 2.**
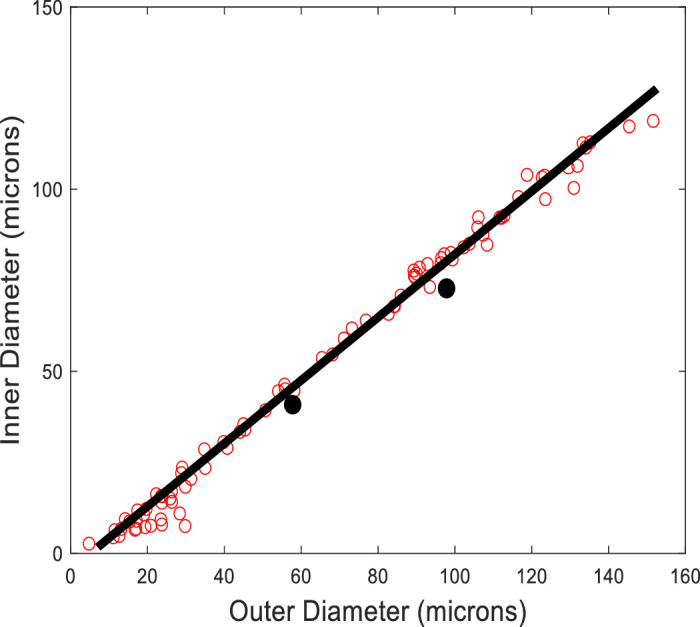
Relationship of inner and outer diameter in young control participants. The inner and outer diameter are highly correlated in young controls (*red open circles*). Based on this relationship, we can predict the expected inner diameter based on the outer diameter (*black line*). The *black closed circles* represent vessels that have deviated from this relationship. For a measured outer diameter, the ratio of the actual inner diameter to the predicted inner diameter, is used as an index of wall remodeling, which we term the arteriole index ratio (AIR). An AIR of one would be expected for healthy blood vessels and lie on the black line. An AIR less than one, as would be the case with vessel remodeling (wall thickened), would lie below the *black line* (*black closed circles*). The AIR is independent of vessel size and, therefore, we can average the AIR for vessels of a given individual to provide an overall AIR. Control data are from Hillard et al.^18^

The total wall thickness was computed as the sum of the thickness of each wall and the WLR was computed as:

$$\begin{array}{c} WLR=\frac{OD-ID}{ID}. \end{array}$$

Perfusion maps were utilized to confirm correct identification of the wall and lumen edges. Values were imported into Microsoft Excel (Microsoft, Redmond, WA, USA), and the arteriolar index ratio (see below) was calculated for each vessel segment. Only arteriole segments with an outer diameter of 50 µm or less were included and 274 arteriole segments (203 from participants with DM and 71 from control participants) were included in the final analysis.

#### Axial Length Correction

All measurements were corrected for retinal magnification based on individually measured axial length (AL).^18^ In three participants with DM, there was no AL measurement available due to equipment failure during their visit. For two of these participants, we calculated the estimated AL based on the spherical equivalent (SE) of their refractive error using the following equation:

$$\begin{array}{c} AL=23.45-0.39\times SE \end{array}$$

derived from a linear model.^36^ For the remaining participant, where no data on refractive error were available, we assumed an AL of 24 mm (the average AL used in our calculations).

### Arteriolar Index Ratio Computation

Because the wall thickness covaries with vessel size in the retina,^18^ we developed a metric of arteriolar remodeling that serves as an index of normality that we refer to as the AIR. This novel metric was originally utilized by Hillard and colleagues to predict the expected inner diameter based on the outer diameter when analyzing wall remodeling in a hypertensive population.^18^ Briefly, using a linear model, Hillard and colleagues found that there was a strong linear relationship between inner and outer diameters of retinal arterioles in young normal participants (r^2^ = 0.989; see Fig. 2). Deviations from this linear relation represent an estimate of the impact of a given disease condition on a vessel. In this study, we computed the ratio of the actual inner diameters to the predicted inner diameter to compute an AIR for each vessel segment. Thus, if the ratio is 1, there is no deviation from the expected inner diameter for a vessel of that outer diameter based on young normal participants.^18^ This approach allowed us to examine changes based on individuals, removing bias based on the varying number of vessels measured across subjects, and can provide a global measure of the arteriolar deviation from controls for each study participant, providing an estimate of how much arteriolar remodeling that individual has undergone by averaging the AIR for all vessels in an individual to give what we refer to as the iAIR. Comparing the iAIRs for all subjects for each group we can then compare groups where each subject is counted once regardless of differences in the number of vessels measured for a given subject.

### Statistical Analysis

All statistical analyses were performed using IBM SPSS Statistics for Windows Version 27.0 (IBM Corp, Armonk, NY, USA). Age differences between the control participants and participants with DM were compared using an independent *t*-test. Gender difference between the two groups were compared using the Pearson χ^2^ test. A *P* value of 0.05 was used for all comparisons except those made with the individual vessel segments.

#### Individual Vessel Segments

For all vessel segments, we compared the difference in means of the inner diameter, outer diameter, wall thickness, WLR, and the AIR between the control and participants with diabetes groups using independent *t*-tests. Because we had 5 planned comparisons for structural measurements of the same vessel segments, we used the Bonferroni correction to establish a significant *P* value of 0.01 for these *t*-tests. When Levine's test yielded unequal variances between the two groups, the Welch's *t*-test was used.

#### Analysis of Individuals Within Groups

To analyze the groups on an individual basis, we averaged the AIR for all arteriole segments measured within an individual as described above to compute the iAIR for each participant. We then used an independent *t*-test to compute the difference in means of the iAIRs between the control groups and the group with diabetes. We then performed 1-way ANOVAs and Tukey post hoc comparisons to analyze the effect of diabetes with and without DR, and/or NPDR on the iAIRs among the control group, diabetes without DR groups, and the diabetes with NPDR groups. Finally, we analyzed the linear relationship among iAIR and age, the iAIR and duration of DM, and the iAIR and central macular thickness.

## Results

Ten participants with DM were excluded due to either poor image quality (*n* = 7), retinopathy due to anemia (*n* = 1), systemic vasculitis (*n* = 1), or concurrent glaucoma (*n* = 1). Twenty-six participants with DM (10 participants with type 1 DM and 16 with type 2 DM) were included in the final analysis and ranged in age from 24 to 79 years (average age of 54 ± 13 years). In our participants with DM, 11 participants had no DR and 15 had DR. Out of the 15 that had DR, 13 of these participants had NPDR, whereas 2 had PDR and 6 participants with DM had some degree of macular edema that did not significantly impact the quality of AO images used in this study. Two control participants were subsequently excluded due to Von Willebrand disease (*n* = 1) or the presence of microaneurysm-like lesions (*n* = 1) on clinical examination. Twenty-two control participants were included in the final analysis, and they ranged in age from 23 to 68 years (40 ± 16 years). All participants included in the study had visual acuities of 20/25 or better. The participants included in the final analysis are summarized in Table 1.

**Table 1.** Characteristics of Participant Groups

	Control	Diabetes	*P* Value
Number of participants and eyes	22	26	
Number of vessel segments	71	203	
Mean age, y	40	53	0.03*
Age range, y	23–68	24–79	
Females, *n* (%)	11 (50.0)	11 (42.3)	0.59

### Differences in Structural Measurements of Arteriole Segments

The use of the semi-automated MATLAB software provided an efficient and useful tool in the quantification of vessel fine wall structure that can be used on curvilinear vessels. Using multiply scattered light from numerous channels allowed us to choose the image with the best contrast for vessel wall structure which varies based on direction and orientation of the aperture and the vessel. It also allowed for the detection of branches in different directions for optimal selection of arteriole segments without branches.^19^^,^^35^

#### Variability in Measurements

Interobserver reliability was measured by having a second expert grader (author T.J.G.) measure each wall thickness, the ID, and OD of 10 random vessels using our semi-automated software. For total wall thickness, all measurements between the two graders were within 1 µm and the intraclass correlation (ICC) was 0.93. For the ID and the ODs, the ICC was 0.98. Therefore, only the measurements from the first grader (author K.A.S.) were used in the final analysis.

The average length of the vessel segments measured was 101 µm in participants with diabetes and 93 µm in the control participants. The differences in means of the structural parameters are summarized in Table 2.

**Table 2.** Structural Remodeling in Arteriole Segments

	Control	Diabetes	*P* Value	Cohen's D
Number of vessel segments	71	203		
**Structural measurements**				
	Mean ± SD	Mean ± SD		
Inner diameter (ID) (µm)	18.6 ± 15.2	17.2 ± 6.9	0.13	0.21
Outer diameter (OD) (µm)	27.4 ± 8.1	27.2 ± 7.8	0.89	0.02
Total wall thickness (WT) (µm)	8.8 ± 1.6	10.1 ± 2.1	< 0.001*	−0.66
Wall-to-lumen ratio (WLR)	0.53 ± 0.19	0.67 ± 0.27	< 0.001*	−0.55
Arteriolar index ratio (AIR)	1.03 ± 0.11	0.95 ± 0.11	< 0.001*	0.82

SD = standard deviation.

#### Wall Thickness

**Figure 3.**
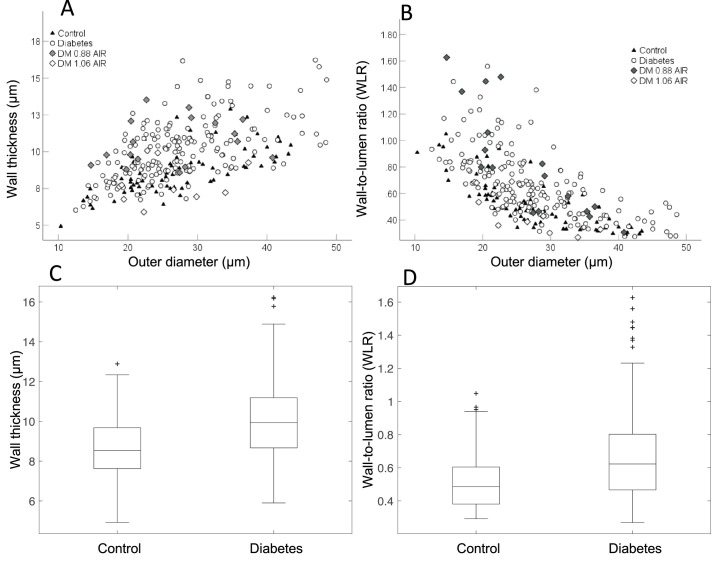
Wall thickness (WT) and wall-to-lumen ratio (WLR) of vessel segments. WT (**A**) and WLR (**B**) plotted as a function of outer diameter in control participants (*black triangles*) and participants with diabetes (*open circles and diamonds*). In both groups, the WLR is inversely related to the outer diameter. On average, the WLR is increased in diabetes (*P* < 0.001). However, the data for vessel segments in participants with diabetes is broader and generally increased compared to controls. The *diamonds* represent two individuals with diabetes. The *gray diamond* is a diabetic individual with a large change in individual AIR (iAIR; 0.88) compared to control participants (mean iAIR of 1.02). The *open diamond* is data from a diabetic individual with an iAIR (1.06) more similar to the mean iAIR in our controls (1.02). The individual vessel segments for the participant with an iAIR of 0.88 shows increased WLRs compared to control data especially for arteriole segments < 25 µm. The diabetic participant with an iAIR of 1.06 has lower individual WLR values. However, in both participants, there is still variation for individual vessels and an overlap with control data. Box plots of wall thickness (**C**) and WLR (**D**) show the significant increase in the arterioles’ segments from participants with diabetes.

The outer and inner diameters of the arterioles measured were similar between groups (*P* = 0.13 and 0.89; see Table 2). Although overlap exists between the groups, the wall thickness was significantly increased in the arteriole segments of the participants with diabetes (*P* < 0.001, Cohen's d = -0.66; Figs. 3A, 3B). Figure 3A also highlights individual data for two individuals with DM, one with near normal and the other more deviated average iAIRs (as discussed below).

#### Wall-to-Lumen Ratio

Like wall thickness, the WLR on average was significantly increased in the vessel segments in participants with DM (*P* < 0.001, Cohen's d = −0.55; see Figs. 3B, 3D). In both groups, the WLR was inversely related to the OD with overlap between the two groups (Fig. 3B). However, the higher WLR generally belonged to arteriole segments from the diabetes group and the increase is especially evident for arteriole segments < 25 µm in outer diameter. The same two participants with DM and different iAIRs are also indicated in Figure 3B using the same symbols as in Figure 3A.

#### Arteriolar Index Ratio

For individual vessel segments, the arteriolar index ratio was increased for participants with diabetes (*P* < 0.001) with a strong effect size (Cohen's d = 0.82; see Table 2).

### Differences in Individuals Within Groups

**Figure 4.**
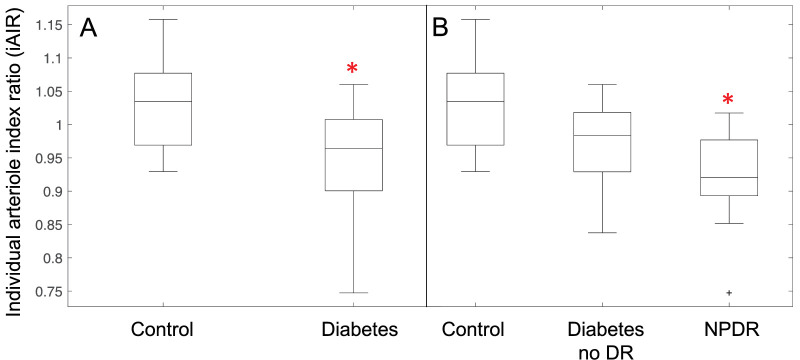
Box plots showing the arteriolar index ratio (AIR) for individuals in each group. Panel (**A**) The difference in individual AIR between control participants and all participants with diabetes groups. Panel (**B**) The difference in individual AIR between control participants, participant with diabetes without diabetic retinopathy (DR), and participants with DM and with non-proliferative diabetic retinopathy (NPDR). In Panel (**B**) the two participants with proliferative DR were excluded. *Significantly different compared to the control group.

The iAIR differed significantly between the control and DM groups (*P* < 0.001; Table 3, Fig. 4). The 95% confidence limits based on the control population distribution of the iAIR are 0.90 and 1.14 and 7 out of the 26 participants with DM fell outside this range. The 1-way ANOVA between the control, DM with DR, and DM without DR groups revealed a significant difference between the three groups (F (2, 45) = 9.75, *P* < 0.001). Post hoc comparisons revealed that there was a significant difference in mean iAIR between the control and diabetes with the DR groups (*P* < 0.001, Cohen's d = 1.49) but no measured difference between the control and diabetes without DR groups (*P* = 0.078) nor between the diabetes groups with and without DR (*P* = 0.250). We then excluded the two participants with PDR to ensure they were not skewing the results, and the 1-way ANOVA results were similar and revealed a significant difference among the three groups (F (2, 43) = 11.16, *P* < 0.001). Post hoc comparisons revealed a significant difference between the control group and the group with NPDR (*P* < 0.001, Cohen's d = 1.64) but no measured difference between the control group and the DM without DR group (*P* = 0.074) nor between the groups with DM without DR and with NPDR (*P* = 0.132).

**Table 3.** Differences in Average iAIR Between Groups

	Comparison				Effect Size
	Control	Diabetes		*P* Value	Cohen's D
Number of participants	22	26			
iAIR (mean ± SD)	1.02 ± 0.06	0.95 ± 0.07		< 0.001*	1.17
	Controls	Diabetes without DR	Diabetes with DR		eta-squared
	
Number of participants	22	11	15		
iAIR (mean ± SD)	1.02 ± 0.06	0.97 ± 0.07	0.93 ± 0.07	< 0.001*	0.302
	Controls	Diabetes without DR	Diabetes with NPDR		
	
Number of participants	22	11	13		
iAIR (mean ± SD)	1.02 ± 0.06	0.97 ± 0.07	0.932 ± 0.07	< 0.001*	0.342

SD = standard deviation; DR = diabetic retinopathy; NPDR = non-proliferative diabetic retinopathy; iAIR = individual arteriole index ratio.

In Figures 3A and 3C, we have also shown how the vessel segments within 2 different individuals with DM vary based on the iAIR. The first individual shown (DM iAIR 0.88) has a more deviated iAIR compared to the second individual (DM iAIR 1.06). In general, the participant with the iAIR of 0.88 had increased wall thickness, WLR, and AIR of their vessel segments compared to controls. The individual with the iAIR of 1.06 had arteriole segments more like controls. However, both participants still had overlap of structural measures with controls.

In the participants with DM, there was no significant linear relationship between iAIR and duration of diabetes (R = −0.19, *P* = 0.39) or with central macular thickness (R = 0.11, *P* = 0.59). One participant was excluded from the analysis with central macular thickness as there was no OCT data available for the day of their AOSLO imaging session. There was not a significant linear relationship between iAIR and age in the control group (R = −0.064, *P* = 0.776). A very weak, but not significant, relationship was observed between age and iAIR in the DM group (R = −0.282, *P* = 0.162).

## Discussion

We measured the structural parameters of retinal arteriole segments using a semi-automated technique at the level of small retinal arterioles. Our analysis shows that DM induces significant remodeling to the wall structure in small retinal arterioles (≤ 50 µm). The remodeling is seen in various structural metrics of the arteriole segments, including increased wall thicknesses and WLRs, and in AIRs that are deviated from what is expected based on a control population. Increased wall thickness and WLRs are consistent with the previous literature of remodeling in resistance vessels due to DM and prediabetes in both subcutaneous and retinal arterioles,^5^^,^^10^^,^^21^^,^^23^^,^^26^^,^^37^ but we are now showing that these findings occur even in the small retinal arterioles. More importantly, the changes in structural remodeling are also evident when we analyze that data for each vessel, and then compute an iAIR's for an individual. Because the vascular morphology, and thus the size of vessels varies widely across the retina, it is not possible to simply pick identically sized vessels across patients. The iAIR, by assigning a normalized index to each vessel, allows us to then calculate the iAIR for each participant, decreasing the impact of covariation of wall thickness and vessel diameter and possibly providing a measure of either systemic or retinal prognosis for the individual patient.

The degree of wall remodeling in small retinal arterioles due to DM has not been widely studied. However, in a hypertensive population, a more marked increase in structural remodeling was observed in retinal arterioles between 10 and 50 µm as compared to larger retinal arterioles^18^ suggesting that this size arteriole, which includes precapillary arterioles, may be more sensitive to vascular remodeling than vessels of a larger caliber. With respect to DM, these small arteriolar changes may be related to impaired autoregulation and loss of myogenic control early in DM due to pericyte loss at the capillary level in addition to other alterations to the vessels arising from hyperglycemia. Based on this study and other studies,^5^ remodeling is present in both large and smaller retinal arterioles and future work should investigate whether this remodeling is present earlier in the course of diabetes and is of greater magnitude in the small arterioles.

With AOSLO, the primary structure visualized and measured is likely the sum of the vascular smooth muscle cells (vSMCs, the largest component), basement membrane, and endothelial cells. Little is known regarding the impact of diabetes on the retinal vSMCs. In diabetic canines, vSMCs are selectively lost.^38^ In type 2 diabetes, the increased MLR is typically attributed to hypertrophic remodeling where the vSMCs become hypertrophic and/or hyperplastic.^8^^,^^10^^,^^13^ Therefore, the changes we observed to the WLR and wall thickness in this study are also likely attributable to changes to the vSMCs as the other wall components are very thin.

WLR is an important biomarker for vascular disease and is associated with increased cardiovascular risk.^8^^,^^39^ In the retina, the WLR is inversely related to the outer diameter as demonstrated by Hillard et al.^18^ for both control participants and participants with hypertension. This dependence is evident in our controls and participants with diabetes even though we restricted the range of outer diameters (< 50 µm) of arterioles measured in this study (see Fig. 3B). Thus, despite this being an important way to evaluate arteriolar remodeling, WLR is not independent of vessel size in the retina. The use of the AIR, which is a ratio of the predicted ID based on the OD and the actual measured ID derived from a healthy group of young controls^18^ allowed us to decrease the impact of any sampling biases on our conclusions by comparing actual measurements for vessels of different sizes to the expected sizes. Thus, this ratio provides an index of normality independent of OD size for individual vessels.

Although significant differences in wall thickness and WLR were present, the difference in the AIR had a stronger effect size. Thus, the AIR may be a stronger indicator of the amount of vessel remodeling in each arteriole, especially as we can control for variability in sampling vessels of multiple sizes in the retina. Furthermore, the iAIR could be calculated for each individual and the median iAIR of the control group was near 1 (see Table 3, Fig. 4) reinforcing the ability of the AIR to capture an estimation of the arteriole properties in normal individuals other than the young control group in the Hillard et al. study^18^ and thus a better vascular biomarker of vessel remodeling. When we analyzed between groups of individuals using the iAIR, we found the mean of the iAIR was significantly deviated in our participants with diabetes compared to controls with a strong effect size (Cohen's d = 1.17). This indicates that in addition to the structural changes from arteriole segments in diabetes, we find that the arterioles of this caliber are remodeled across the retinae of individuals with diabetes. As 7 out of the 26 individuals with diabetes had iAIRs outside of the lower limit of the 95% confidence limits of the normal control population, the iAIR should be continued to be investigated as a potential biomarker. Further division of our participants with diabetes into three groups by separating the individuals with diabetes with and without retinopathy (excluding those with PDR), we found that the group with NPDR had a significantly different mean iAIR compared to controls. This finding is consistent with the expectation that it would be likely to find increased remodeling of the retinal arterioles when clinically detectable lesions are present but still early in the DR spectrum. As it captures changes at a more cellular level compared to the detection of clinical lesions, the iAIR may provide insight into how remodeling or reverse remodeling may occur due to therapeutic interventions in the future. In addition, the difference in remodeling due to DM and HTN and HTN alone should be assessed in the retinal arterioles in the future. We have begun preliminary analyses to answer this question, and thus far the remodeling between these groups is similar, but we are limited in the number of small arterioles and participants with HTN only to draw any conclusions.

Although structural metrics of the retinal arterioles differed between the diabetes and control groups, there was a large amount of overlap. This is not surprising, because participants with diabetes are often highly variable in their systemic control, duration of disease, therapeutic interventions, and overall response to diabetes.^40^ Whereas it could be that the variability in our vessel metrics differed widely between individual blood vessels, the iAIR indicates that a large amount of the variability was related to the individual. In Figure 3, we highlight vessel measurements from 2 individuals with very different iAIRs: one individual with a more deviated iAIR of 0.88 and the second individual with an iAIR of 1.06 that is more like that of the average control iAIR of 1.02 in this study. Data from these 2 individuals are plotted in Figures 3A and 3B. The individual with the iAIR of 0.88, in general, has thicker walls and increased WLRs and, at the time of imaging, was a 42-year-old man with newly diagnosed with type 2 DM. Clinically, this individual had mild NPDR and self-reported that their last glycosylated hemoglobin (HbA1C) was 9 about 2 weeks prior to the imaging session. They also had controlled hypertension. Conversely, the individual with the iAIR of 1.06 was a 50-year-old woman, had no DR, and a reported HbA1C of 5.6. This individual had a 27-year history of type 2 DM and was extremely well controlled since receiving their initial diagnosis and treated with metformin and liraglutide. Additionally, this individual was also being treated with lisinopril despite having never received an official diagnosis of HTN. Although no concrete conclusions can be made, it is speculative whether the tight control and good compliance with antidiabetic medications enabled the individual with the iAIR of 1.06 to maintain, or perhaps even reverse, arteriole remodeling within their retina. With systemic hypertensive therapy, it has been shown that reverse remodeling with decreased MLR of subcutaneous arterioles in non-insulin–dependent diabetes mellitus (NIDDM) after 1 year of treatment occurs.^8^ Likewise, decreases in WLR with antihypertensive treatment is also observed in retinal arterioles.^41^^,^^42^ It will be important for future studies to understand the impact of antidiabetic and antihypertension medications on retinal vascular remodeling and the rapidity with which this occurs. This could provide insight as to whether some medications might have a more positive impact on the retinal vascular structure than others in participants with DR or increased risk factors for DR. Last, although the iAIR in the individual with an iAIR of 0.88 was deviated, some individual vessels in this participant are similar, or overlap with, that observed in the healthy controls. As this participant did have clinical DM, whereas the current protocol was not designed to address this, investigating the variability within individuals and relationship of remodeled arterioles spatially to local retinopathic changes may provide an opportunity for the AIR to provide both a global (iAIR) and a local metric of retinal health (AIR of individual arterioles). These two participants display the issue of making vascular measurements on a population with a wide range of histories and supports the need for future studies with larger numbers of participants with well characterized medical histories to better determine the risk factors associated with alterations of the microvasculature due to diabetes.

In our study, we included participants with both types 1 and 2 DM of which 76.9% (20 out of 26) also had controlled and pharmacologically managed HTN. Because diabetes is often comorbid with HTN, isolating participants with DM but no history of HTN is challenging. In the retina, HTN and increased blood pressure induce wall thickening and increases in WLRs in the retinal arterioles.^18^^,^^43^^–^^47^ In parallel, the subcutaneous resistance vessels from biopsy suggests that the volume of the smooth muscle cells (wall-cross sectional area) is constant meaning that the lumen has narrowed and the smooth muscle cells have been re-arranged around a smaller lumen indicative of inward eutrophic remodeling.^8^^,^^13^ This thickening of the vascular walls and lumen narrowing of arterioles is a response to the increased blood pressure in HTN and serves to protect the capillary bed.^8^ In DM, hyperglycemia and the upregulation of other growth factors and consequences of the disease have a direct effect on vascular integrity. In non-insulin dependent diabetes without HTN, the volume of the vascular muscle cells (wall-cross sectional area) is increased and the increase in MLR is greater than that observed in individuals with HTN alone.^8^ Thus, it is likely that DM itself induces morphological changes to the arterioles independent of HTN. However, the highest degree of arteriole remodeling was observed in individuals with both NIDDM and HTN indicating that there is a synergistic effect of these comorbidities on the remodeling of subcutaneous arterioles.^8^ Given that most of the participants in this study also had HTN, we presume that the remodeling observed is due to DM but also has a component of remodeling from HTN in the participants with both comorbidities. As the risk of DR development and progression increases with uncontrolled concomitant HTN, this further supports the hypothesis that there is a synergistic effect of DM and HTN on vascular remodeling within the retina. We have begun preliminary work to address this question and recognize that further investigation into this hypothesis should be performed in the retinal arterioles in the future to determine the relative contributions of each to the remodeling.

**Figure 5.**
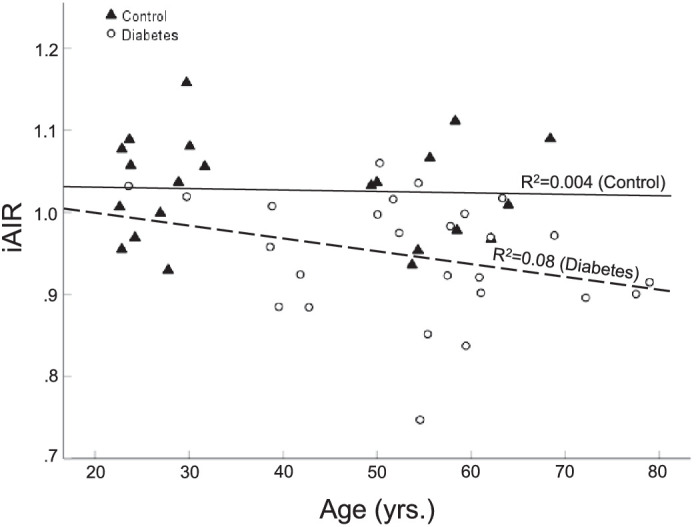
Linear relationship of the average individual arteriole index ratio (iAIR) and age. In the control group, there is no correlation between age and iAIR (*solid black line*). In the group with diabetes (*dashed line*), there is a weak, but not significant, negative correlation of iAIR with age.

Although there are differences in the interaction of age and wall remodeling,^5^^,^^13^ in the current study, there was no age effect on iAIR (Fig. 5). In the participants with DM, there was a weak interaction with age, but age is a strong covariant of DM changes and thus the small age difference between the DM and control populations is unlikely to be related to a change in iAIR with age.

There are several limitations to this study. First, this was a retrospective cohort study which may be prone to selection bias. To minimize this, only the participant number was used when analyzing the AOSLO blood vessel remodeling data. The DR grading and review of the clinical examination were performed after all vessels were graded. Second, ocular clarity can impact image quality during our AOSLO imaging sessions and tear film quality can be especially compromised in participants with diabetes. This could potentially cause issues with participants with better image quality skewing the data. However, this impact would have been most profound in our analyses of the structural parameters of vessel segments. Because we still found a significant increase in the average iAIRs, this type of sampling bias is unlikely to have impacted our results. In addition, because the use of an individual specific index of abnormality was developed, we can weight each participant equally in the overall analysis. Third, only small retinal arterioles were included in this study. Although we suspect the smaller retinal arterioles are impacted earlier and more significantly in DM, future investigations should assess the difference in a similar manner to Hillard and colleagues^18^ in a population with diabetes. Fourth, a large fraction of our participants with DM also had HTN and were taking systemic antidiabetic and antihypertensive medications which may impact vascular remodeling. Last, DM is an extremely variable disease, and this is represented in the spectrum of vessel remodeling detected in our group of participants with DM. In the future, monitoring these participants over time with blood testing at the time of imaging, perhaps in a more integrated healthcare facility, is important to understand how these vascular alterations occurs with the spectrum of disease. Unfortunately, we did not have these capabilities and the variability in the timing of the self-reported metrics we had like last glycosylated hemoglobin, did not enable us to analyze this relationship.

In summary, we have used AOSLO multiply scattered light imaging to quantify the arteriolar structural remodeling in small retinal vessels due to diabetes. Our findings suggest that, like larger retinal arterioles, the wall thickness and WLR in vessels smaller than 50 µm increases in diabetes. We have also shown that the use of a normalizing index to control for vessel size (AIR) for analysis of both the vessel segments and to compute an average AIR for individuals (iAIR), provides a robust measure of the remodeling occurring in these vessels that is not confounded by the impact of selection bias due to vessel size. Because many of the individuals with diabetes are outside the lower 95% confidence limit of the normal control population, it is an excellent candidate biomarker to monitor vascular remodeling in the retinal microvasculature and should be further explore its relationship to other complications of DM. Using an index of normality such as this is an excellent candidate biomarker to better monitor structural remodeling in the retinal vessels in diabetes with the potential to apply to numerous pathological conditions that affect the retinal microvasculature, systemic vascular disease, and evaluate vascular responses to current and new therapeutic interventions. This is especially important as anti-VEGF therapy is currently being investigated as a means of intervention in NPDR to prevent disease progression^48^^,^^49^ and this could provide insight to cellular level responses. Likewise, such a measurement could be used to evaluate response of the retinal vasculature to systemic interventions and impact on arteriolar remodeling as new diabetic interventions may have an impact on vascular structure in addition to classical interventions focusing on glycemic control. It is possible that a similar index could also be developed for vessels elsewhere in the body. Future investigations should include the measurement of small and large vessels within the same individuals; the relationship of these wall/lumen changes to alterations in blood flow and retinal function; and longitudinal monitoring of the effects of age, sex, blood pressure, disease duration, and treatment on these findings.
